# Improving Sleep Quality to Enhance Athletic Activity—The Role of Nutrition and Supplementation: A Mini-Short Review

**DOI:** 10.3390/nu17111779

**Published:** 2025-05-24

**Authors:** Jonathan Fusi, Giorgia Scarfò, Raul Di Silvestro, Ferdinando Franzoni

**Affiliations:** Department of Clinical and Experimental Medicine, University of Pisa, 56126 Pisa, Italy; jonathan.fusi@med.unipi.it (J.F.); giorgiascarfo91@gmail.com (G.S.); rauldisilvetro00@gmail.com (R.D.S.)

**Keywords:** sleep, exercise, insomnia, melatonin, nutrition, antioxidant, caffeine

## Abstract

Sleep is a fundamental part of life for all living beings. The propensity to fall asleep is regulated by a circadian rhythm, which controls the secretion of the hormone melatonin. Melatonin secretion is linked to the light and dark phases of the day/night cycle. Getting proper sleep is an essential part of a healthy lifestyle. Scientific evidence shows that sleeping less than 7 h per night, or as little as 2 h per night, is a cardiovascular, metabolic, and cerebral risk factor. In addition, the role of sleep is fundamental for the recovery phase for athletes. Nutrition, supplementation, and exercise can greatly support the quality and quantity of sleep. They can have positive effects on sleep through specific physiological and biochemical responses. The objective of this short review is to critically investigate the possible sleep benefits of nutrition, exercise, and supplementation and to discuss further directions for research in this area.

## 1. Introduction

Sleep is a global state of immobility with greatly reduced reactivity to environmental stimuli [1]. It is regulated by circadian or ultradian rhythms and characterized by rapid reversibility [2]. Sleep is an essential biological condition for the individual, and when there is a decrease in the amount of sleep had, the organism tries to recover the amount lost, leading to a rebound effect [1].

Sleep is responsible for many functions, from energy functions to immune and thermoregulatory functions and cardiovascular health. All these conditions are necessary for normal homeostasis [3].

The study of sleep was made possible using electroencephalography, which allows us to divide sleep into two subcategories: non-rapid eye movement (N-REM) sleep and rapid eye movement (REM) sleep [4]. In the initial phase of sleep, brain deactivation is observed with a loss of awareness and a subsequent slowing of brain waves, a condition referred to as N-REM sleep. Later during sleep, there is the REM phase, in which eye movements and small involuntary muscle contractions are observed [3]. These two phases alternate regularly for approximately 90 min. This alternation shows that there is a high degree of specific oscillation between these two phases [4].

Neurologically, sleep is regulated by two macroregions: the hypothalamus, which is involved in circadian control, and the brainstem and forebrain nuclei, which project neuromodulator signals to the basal ganglia, amygdala, thalamus, hippocampus, and cortex. These two macroregions underline the alternation of the NREM and REM phases [5,6].

As described above, the quantity and quality of sleep are important for the proper functioning of many activities, from cardiovascular to metabolic and immunoregulation [4]. Adults should get around 7 h of sleep per night [7].

Sleep deprivation has been shown to be related to the onset of numerous diseases, including obesity, hypertension, and cardiovascular diseases [8,9].

Sleep deprivation can also lead to alterations in hunger and appetite. Subjects with sleep deprivation are inclined to consume more snacks that are unhealthy for metabolism. This increased intake of snacks is probably due to an altered insulin and leptin-ghrelin profile that has been observed in sleep-deprived individuals [10,11,12,13,14]. All the alterations in cardiovascular, metabolic, and immunological risk, as well as a decrease in cognitive functions, could be induced by the hormonal and circadian rhythm alterations caused by sleep deprivation [15,16].

Therefore, improving lifestyle habits such as proper nutrition and physical activity could induce significant improvements in the quantity and quality of sleep.

The purpose of this review is to provide some quick and useful information on the interaction between sleep, sport, and food.

## 2. Sleep and Exercise Recovery

Sleep is of huge help to athletes, but in some cases, where it is poorly managed, it can alter athletes’ performance and lead to a high risk of injury [17]. Compared to a sedentary or averagely active adult, an athlete may require a longer period of sleep. In fact, among countless alterations, sleep deprivation may result in a decrease in growth hormone accompanied by an increase in circulating cortisol [18]. This is due to the increase in pro-inflammatory cytokines, and in addition to this, a slowdown in the recovery phase may also occur, thus leading to a risk of incurring injuries and decreased performance. Currently, it appears that sleep deprivation can affect various activities, such as endurance training, sprint training, and memory skills, in a specific way [19]. Fullagar et al. (2015), in their study, observed how sleep deprivation results in an increase in perceived exertion that can induce a decrease in endurance performance [20]. In this regard, the study carried out by Oliver et al. (2009) [21] seems to be very interesting and is able to shed light on this issue. In his study, he showed that a night of sleep deprivation reduced endurance performance, with limited effects on either cardio-respiratory or thermoregulatory function [21]. While on endurance performance the data are in good agreement with each other, the findings in the literature are in stark contrast with each other regarding the responses to sleep deprivation on sprinting abilities. In fact, while there is a decrease in intermittent sprint times after 30 h of sleep deprivation, this result was not observed in another study, in which no alteration was found regarding 40 m sprint activities in subjects after 64 h of sleep deprivation [22]. As is possible to imagine, not only is the physical aspect understood as a cardiopulmonary response or resistance to fatigue or strength, but sleep absence or deprivation can result in alterations in more technical activities. Edwards et al. (2009) observed a decrease in accuracy in dart throwing and serving in tennis players in athletes after an incomplete night’s sleep of 4–5 h [23].

When analyzing research data in relation to physical activity, it is always difficult to compare them with one another, as numerous variables often change, including the type of physical activity, recovery times, and the age of the subjects tested. Certainly, sleep deprivation can lead to alterations in performance, as has been shown [24]. In the literature review conducted by Craven et al. (2022) [24], it is observed that acute sleep deprivation negatively influences performance the next day. In contrast, however, the incidence is related to the type of sport and performance required of the athlete. Probably, the negative changes in relation to sleep loss are to be found in several factors, from physiological ones (such as heart rate) to technical ones (technical gestures). In fact, the increase in certain hormones such as cortisol could affect performance by impairing the ability to concentrate and manage heart rate [24].

## 3. Nutrition and Sleep Quality

Nutrition can influence sleep quality and quantity, and negative changes in sleep duration and patterns are often associated with an unhealthy diet [22,25].

Caffeine is among the nutrients that most influence sleep. Caffeine intake, especially in the evening, negatively affects the quantity and quality of sleep [26]. Caffeine, in fact, acts as a stimulant, since it can cross the blood–brain barrier and antagonizes sleep-inducing adenosine receptors (A2AR) [27]. This function occurs following acute caffeine intake, while the chronic effects of caffeine have not yet been fully elucidated in the scientific literature [28,29].

In contrast, melatonin positively impacts sleep patterns [30], as well as being an important regulator of the sleep–wake cycle, through its interaction with two G-protein-coupled receptors (MT1 and MT2). In addition to its exogenous intake, melatonin is regularly produced by the pineal gland [31]. Macronutrients, including carbohydrates, fatty acids, and amino acids, can influence sleep [32].

As far as carbohydrates are concerned, their effects on human metabolism and sleep are closely dependent on their glycemic index [32,33,34]. Specifically, Afaghi et al. (2007) observed that the intake of foods with a high glycemic index 4 h before sleeping caused a decrease in the quantity and quality of sleep [35]. This was later confirmed by Gangwisch et al. (2020) [36]. The authors suggested that in postmenopausal women there was a strict relationship between insomnia and high glycemic index carbohydrate intake [36].

In this regard, it is necessary to clarify that drowsiness after a meal is only the consequence of a series of physiological mechanisms in response to the post-prandial hyperglycemia: particularly, a sudden insulin release causes a drastic decrease in plasma glucose concentration around ∼3.8 mmol/L [37], followed by the secretion of counterregulatory hormones, including adrenalin and noradrenalin [38,39].

Conversely, low-glycemic-index food consumption before bedtime positively influences sleep in both sedentary and physically active individuals [40,41]. Sleep is fundamental in athletes and represents “functional recovery”, which is a crucial period in which pulsatile hormone secretion, muscle growth, and muscle fiber repair phenomena take place. However, there are still few studies about the relationship between nutrition and sleep in athletes [40,41].

Vlahoyiannis et al. (2018) observed that a high-glycemic-index meal after a training session could induce an improvement in sleep quality, with a reduction in sleep onset latency [40].

Daniel et al. (2019) investigated the differences between a high-glycemic-index meal and a low one before going to sleep [41]. The quality and quantity of sleep was assessed by actigraphy. The researchers did not find alterations in salivary cortisol or melatonin concentration between these two kinds of meals, even if the caloric intake and daily food type were important variables in terms of sleep quality and quantity.

Both the aforementioned studies seem to be in contrast with what happens in sedentary individuals, maybe because of athletes’ different metabolic mechanisms and biochemistry: particularly, carbohydrates are the main fuel source for athletes [42], and the depletion of muscle glycogen post-exercise alters the insulin response [43]. The intake of a high -glycemic-index meal could restore muscle glycogen, improve fatigue, and increase blood tryptophan levels [44], which are a precursor of melatonin [45]. The above-mentioned study data may be at odds with each other. Probably, the main problem is related to the type of diet and type of exercise and the timing of intake of the carbohydrate-rich meal. Certainly, the replenishment of high-glycemic-index carbohydrates, taking advantage of the high insulin response induced by exercise, turns out to be instrumental in improving recovery [42,43]. In terms of improving sleep quality, the data may be contradictory in that many athletes prefer not to eat very large meals before going to bed, while others require rich and plentiful meals to fall asleep better. Therefore, the role of the physician, dietician, and nutritionist in structuring highly personalized nutritional plans becomes essential [46] to improve both the muscle glycogen replenishment phase and the sleep phase.

## 4. Supplements and Sleep Quality

### 4.1. Caffeine

Caffeine is a widely consumed stimulant substance [47]. It exerts its action by binding to A1 and A2 adenosine receptors and acting as an antagonist [28]. Due to its stimulant properties, it has been proposed that it can improve physical performance, even with ambiguous results [48].

The first studies on the effects of caffeine date back to 1910. Over the years, scientific studies have highlighted the relationship between caffeine and sleep, and its acute and chronic capacity to affect the circadian rhythm [28]. In a murine model, chronic caffeine consumption induced a decrease in the resting phase [49]. Weibel et al. (2021) evaluated caffeine intake (10 days, 3 × 150 mg/day) in twenty healthy young subjects, demonstrating that caffein worsened the REM phase [50]. This was in line with previous studies, in which caffeine intake 30 min before bedtime provoked insomnia and altered the sleep–wake rhythm [51,52].

### 4.2. Magnesium

Magnesium (Mg^2+^) is the intracellular cation involved in several biological functions and plays an active role in at least 600 enzymes [53], as well as in DNA and RNA production. Approximately 25 gr of it is found inside the human body [54]. It is predominantly involved in energy production mechanisms, including oxidative phosphorylation [55]. Feeney et al. (2016) recently observed the active role of magnesium in the regulation of circadian rhythm and sleep quality [56]. Hornyak et al. (2004) [57] observed how the administration of 729 mg magnesium (MgO) in alcohol-dependent patients during sub-acute abstinence from alcohol induced better sleep quality. Not only that, they also found a decrease in sleep onset latency [57]. Improvement in sleep quality was also observed in a study by Nielsen et al. (2010) [58] in a population of participants over 51 years of age. It was observed that an intake of 320 mg magnesium/day via a magnesium citrate supplement for 7 weeks induced an improvement in sleep quality [58]. Held et al. (2002) demonstrated how an administration of 729 mg magnesium (MgO) induced an improvement in slow-wave sleep on an EEG [59].

Magnesium can induce positive sleep modifications in elderly individuals through two pathways [60]. In fact, magnesium can act both as an NMDA antagonist and a GABA receptor agonist, and can affect the neuroendocrine system, promoting the release of melatonin and reducing symptoms of insomnia [61,62]. Zhao et al. (2025) [63] investigated the possible association between daily Mg intake and self-reported sleep duration and sleep disorders. In his study, 21,840 subjects (2009–2018) of the National Health and Nutrition Examination Survey (NHANES) were analyzed. The data analyzed showed that in the participants who took oral magnesium supplements, there were no differences compared to the control group in sleep duration or self-reported sleep disorders [63]. On the other hand, however, as Mah and Pitre (2021) suggest, magnesium supplementation is worthwhile for other positive effects in addition to the possible effects it has on sleep, and especially on energy, and was therefore considered essential for athletes [60].

According to the Dietary Guidelines for Americans 2015–2020, daily Mg^2+^ intake should total 320 mg/day for women and 420 mg/day for men [64], with some fluctuations in certain physiological and pathological conditions (e.g., pregnancy; type 2 diabetes).

Unfortunately, there are still few studies on the relationship between magnesium and sleep, and further studies are needed to understand it [60].

### 4.3. Melatonin

Endogenous melatonin secretion is primarily regulated by the pineal gland and secondarily by the retina, as well as the gastrointestinal tract and bone marrow cells [65]. Its circulating levels vary throughout the day from 5 to 200 pg/mL [66]. The main role of melatonin is the modulation of the sleep–wake cycle. In addition, antioxidant and anti-inflammatory properties have been observed [67,68]. Their roles as sleep–wake rhythm regulators have been suggested by several studies, in which subjects suffering from insomnia showed a decrease in melatonin levels [68,69].

Consequentially, melatonin supplementation (1–5 mg/die) has been proposed to improve sleep quality and quantity [65].

Regarding sleep onset latency, instead, results are conflicting [70,71]. Hughes et al. (1998) observed how high doses of melatonin taken 30 min before going to bed improved sleep at night [70].

Vural et al. (2014) investigated optimal melatonin supplement dosages in elderly subjects (0.1 mg to 50 mg/kg) [72]. Based on their data, they found that a low dose of melatonin is needed in older people, since a high dose (3 mg) [71] seems to be associated with prolonged plasma melatonin levels throughout the following day, causing drowsiness [73,74]. The dose–response model proposed by Cruz-Sanabria et al. (2024) showed that taking 4 mg/day of exogenous melatonin is most effective in reducing sleep onset latency (SOL) [75]. Conversely, intake of 3 mg/day was shown to be effective in improving total sleep time (TST). Clearly, the timing of intake could also be decisive in improving exogenous melatonin function. Indeed, it has been shown that taking exogenous melatonin between 1 and 3 h before falling asleep is the most effective [75].

To optimize melatonin levels, it is possible to consume foods containing melatonin, or its precursors, such as bananas, apples, pineapples, and grapes, as well as walnuts, almonds, brown rice, oats, and tomatoes [76,77,78].

### 4.4. Tryptophan

Tryptophan (TRP) is an essential amino acid and is involved in sleep regulation, being a precursor of serotonin (-Hydroxytryptamine; 5-HT) [79] and melatonin. Indeed, 5-HT is converted into melatonin by the arakylamine N-acetyltransferase [80]. Tryptophan metabolism occurs via the kynurenine and serotonin pathways. The dietary intake of tryptophan is essential for the synthesis of niacin [81] and serotonin synthesis [79]. As mentioned above, serotonin synthesis occurs via the 5-HT pathway. In the intestine, serotonin is converted to N-acetylserotonin, which is then converted to melatonin by hydroxyindole-O-methyltransferase [82]. An interesting feature of tryptophan is its ability to cross the blood–brain barrier [83]. Due to being a precursor of melatonin, several studies highlighted the relationship between TRP and sleep [79].

It has been demonstrated that both tryptophan-rich foods and tryptophan supplementation can improve sleep quality in people with insomnia [84,85]. Sutanto et al. (2022) [79] investigated, in their meta-analysis, the role of tryptophan supplementation on sleep quality. Through a detailed literature review, the authors concluded that TRP supplementation at ≥1 g can help improve sleep quality. However, the intake of tryptophan-rich foods such as, for example, milk or cereals, has been always shown to play a positive role in improving sleep [86]. Regarding TRP supplementation, it has been demonstrated that less than 1 g of TRP did not affect insomnia, while an amount greater than 1 g was needed to increase TRP brain levels, with positive effects on the treatment of insomnia [87].

### 4.5. Ashwagandha

Ashwanga, also known as *Withania somnifera*, is a remedy in Ayurvedic medicine. Many studies point to its positive role in regulating stress and cognitive functions [88,89]. In association with its adaptogenic properties, a decisive role of Ashwanga in improving performance has been proposed [90]. A study by Pérez-Gómez et al. (2020) highlights how the use of this Ayurvedic remedy can improve maximal oxygen consumption [91]. In addition, the authors, through a detailed analysis of the literature, also suggest the limits of intake to be functional and safe for health. A range of 330 up to 1250 mg/day appears to be well tolerated and safe [92]. In addition to possible performance improvements, the use of Ashwagandha appears to be of interest in improving sleep quality, probably due to its properties in regulating stress [88]. Langade et al. (2021) [93] investigated the actual effect of using this extract in improving sleep quality. They administered it to 40 subjects to be taken at eight set times and compared the sleep quality parameters (i.e., sleep onset latency) in a control group. The authors found improvements in sleep quality [93]. These data were observed and discussed in more detail in the meta-analysis conducted by Cheah et al. (2021), in which five trials involving 400 adults diagnosed with insomnia were analyzed [94]. From the data obtained, it was possible to observe that treatment with 600 mg/day for 8 weeks resulted in improvements in mental alertness and anxiety. The authors, however, concluded their review of the literature by emphasizing that further studies are necessary to understand the actual efficacy and safety of Ashwagandha supplementation.

### 4.6. Apigenin

Apigenin (i.e., 5, 7, 4-trihydroxyflavone)—a principal ingredient of cirsium japonicum—is one of the most common flavones, which has also been isolated in hypericum perforatum flowers [95]. This compound is widely distributed in many fruits and vegetables, including cabbage, bell pepper, garlic, celery, and guava. Apigenin exhibits neuroprotective effects against oxidative stress by blocking caspase-3 activity and glutamate-induced neurotoxicity by reducing NMDA receptor-mediated responses [96]. In addition to the above-mentioned activities, its role has been found in improving sleep quality in both animal models and humans [97]. In human model studies, chamomile extracts characterized by 1% Apigenin are used. The concentrations of chamomile extracts used in the studies ranged from 220 mg/day to 270 mg/day to 1000 mg/day for at least 4 weeks, observing improvements in mood quality and depressive phenomena [98,99]. Amsterdam et al. (2020) [100] achieved a concentration of 1500 mg/day for eight weeks. In their work, they showed that this concentration is helpful in improving anxiety levels and depressive symptoms [100]. We can therefore briefly conclude that the improvements in sleep induced using chamomile extracts (220-270-1000-1500 mg/day) are to be found in ancillary effects such as a decrease in depression and anxiety and an improvement in oxidative stress [97].

## 5. Role of Exercise in Good Sleep

Regular exercise reduces risk factors such as obesity and hypertension, and has protective effects against cardiovascular diseases and mood disorders [101,102]. According to current guidelines, 150 min of aerobic exercise two times per week of resistance training [103] should be performed. Regular physical activity can also improve sleep quality and quantity [104].

Montgomery et al. (2002) [105] investigated the use of physical activity as a non-conventional therapy for elderly subjects with sleep disorders. The researchers observed that exercise improved sleep duration, onset latency, and the Pittsburgh Sleep Quality Index (PSQI) score [105]. Irwin et al. (2008) showed the effect of 9-week Tai Chi Chih training on sleep quality and demonstrated its ability to improve sleep quality scores [106]. Recently, Sullivan Bisson et al. (2019) [107] investigated the possible role of walking as an intervention to improve sleep quality and duration. The program consisted of a 4-week intervention in which subjects had to walk more than 2000 steps per day and increase by 2000 steps each week, and the group subjected to this program experienced higher sleep durations [107].

The possible mechanisms by which exercise improves sleep quality and duration are neuropsychological and neurovegetative. High-concentration exercise, such as Yoga, Tai Chi, Tai Chi Chih, and stretching, could have anti-anxiety and antidepressant effects [106], in addition to increasing the activity of the parasympathetic nervous system [108,109].

Moreover, regular physical activity can modulate body temperature [105], inflammatory responses, and Brain-Derived Neurotrophic Factor (BDNF) secretion [110,111].

In parallel, exercise also improves sleep through similar mechanisms, both globally and locally [112,113].

## 6. Conclusions

Sleep is a fundamental part of the day for every living organism, during which the body can be rebalanced and cleansed off all daily waste products. Insomnia, or alterations in sleep, can lead to increased inflammation, oxidative stress, and weight alterations (overweight, obesity). Such alterations can affect training and the response to training itself. In fact, incomplete rest can lead to an increased inflammatory state, increasing injury risk and leading to decreased performance.

Nutrition, supplementation, and physical activity can play a key role in modulating the sleep–wake rhythm. A Mediterranean diet characterized by low-glycemic-index foods and tryptophan-rich foods seems to be the best way of life to improve sleep quality and duration. In terms of supplementation and physical activity, the literature is still conflicting, and further investigation is needed to understand the real physiological and biochemical mechanisms involved. It would be interesting to understand whether there is a specific type of physical activity that improves sleep quality (in addition to examples such as Tai Chi), and how much physical activity should be performed before falling asleep. Such a recommendation might also be useful for those in target sports. In fact, one might consider including within a training program a final taper session with exercises to improve sleep and allow for better recovery. Furthermore, given the numerous conflicting data on sports, nutrition, supplementation, and sleep, it would be useful to investigate whether there is an epigenetic correlation between sports, nutrition, and sleep.
